# Metabolic Rewiring and the Characterization of Oncometabolites

**DOI:** 10.3390/cancers13122900

**Published:** 2021-06-10

**Authors:** Diren Beyoğlu, Jeffrey R. Idle

**Affiliations:** Arthur G. Zupko’s Division of Systems Pharmacology and Pharmacogenomics, Arnold and Marie Schwartz College of Pharmacy and Health Sciences, Long Island University, Brooklyn, NY 11201, USA; diren.beyoglu@liu.edu

**Keywords:** oncometabolite, metabolomics, fumarate, succinate, (2*R*)-hydroxyglutarate, (2*S*)-hydroxyglutarate, hypoxia, histone demethylation, DNA demethylation

## Abstract

**Simple Summary:**

Oncometabolites are produced by cancer cells and assist the cancer to proliferate and progress. Oncometabolites occur as a result of mutated enzymes in the tumor tissue or due to hypoxia. These processes result in either the abnormal buildup of a normal metabolite or the accumulation of an unusual metabolite. Definition of the metabolic changes that occur due to these processes has been accomplished using metabolomics, which mainly uses mass spectrometry platforms to define the content of small metabolites that occur in cells, tissues, organs and organisms. The four classical oncometabolites are fumarate, succinate, (2*R*)-hydroxyglutarate and (2*S*)-hydroxyglutarate, which operate by inhibiting 2-oxoglutarate-dependent enzyme reactions that principally regulate gene expression and response to hypoxia. Metabolomics has also revealed several putative oncometabolites that include lactate, kynurenine, methylglyoxal, sarcosine, glycine, hypotaurine and (2*R*,3*S*)-dihydroxybutanoate. Metabolomics will continue to be critical for understanding the metabolic rewiring involving oncometabolite production that underpins many cancer phenotypes.

**Abstract:**

The study of low-molecular-weight metabolites that exist in cells and organisms is known as metabolomics and is often conducted using mass spectrometry laboratory platforms. Definition of oncometabolites in the context of the metabolic phenotype of cancer cells has been accomplished through metabolomics. Oncometabolites result from mutations in cancer cell genes or from hypoxia-driven enzyme promiscuity. As a result, normal metabolites accumulate in cancer cells to unusually high concentrations or, alternatively, unusual metabolites are produced. The typical oncometabolites fumarate, succinate, (2*R*)-hydroxyglutarate and (2*S*)-hydroxyglutarate inhibit 2-oxoglutarate-dependent dioxygenases, such as histone demethylases and HIF prolyl-4-hydroxylases, together with DNA cytosine demethylases. As a result of the cancer cell acquiring this new metabolic phenotype, major changes in gene transcription occur and the modification of the epigenetic landscape of the cell promotes proliferation and progression of cancers. Stabilization of HIF1α through inhibition of HIF prolyl-4-hydroxylases by oncometabolites such as fumarate and succinate leads to a pseudohypoxic state that promotes inflammation, angiogenesis and metastasis. Metabolomics has additionally been employed to define the metabolic phenotype of cancer cells and patient biofluids in the search for cancer biomarkers. These efforts have led to the uncovering of the putative oncometabolites sarcosine, glycine, lactate, kynurenine, methylglyoxal, hypotaurine and (2*R*,3*S*)-dihydroxybutanoate, for which further research is required.

## 1. Introduction

The description of the structure of DNA reported by Watson and Crick in 1953 [1,2] and the discovery of cellular oncogenes by Varmus and Bishop in 1976 [3] not only led to the award of the Nobel Prize to each of these scientists, but also had a secondary and little discussed impact on cancer biology. These monumental research milestones deviated cancer research away from small molecules to the macromolecular and genetic level. Up until these turning points, there had been considerable research activity into the role that cancer has on cellular metabolism and the effects of cellular metabolism of environmental chemicals on cancer causation. After Watson and Crick, the field of biochemistry underwent a transformation away from intermediary metabolism, swiftly culminating in the discovery of tRNA [4], mRNA [5,6] and ribosomes [7]. The pioneering work of Otto Warburg in the 1920s on altered metabolic pathways in cancer cells was largely to remain unrecognized for 80 years. Following the oncogene discovery, the role of chemical metabolism in carcinogenesis was gradually to be eclipsed by oncogenes, tumor suppressor genes and cell signaling. As Weinberg [8] put it, “The virologists [were] emboldened by the discovery of new animal cancer viruses and their new theories… those who had spent their lives researching chemical carcinogens became increasingly demoralized, seeing no clear way to advance their work”. Fortuitously, today, cell metabolism has returned to the center stage in cancer research. As Wishart has asked, is cancer a genetic disease or a metabolic disease? He argues that decades of cancer analysis have uncovered nearly 1000 known cancer-associated genes (~250 oncogenes and ~700 tumor suppressor genes) and that typically two mutations in each of these genes would lead to >1 million cancer genotypes, making the genetic fingerprinting of cancers for personalized oncology a formidable task [9]. On a more optimistic note, he continues that most oncogenes and tumor suppressors play fundamental roles in cellular metabolism involving a limited number of pathways. He concludes that while “cancer as a genetic disease looks to be impossibly complex, cancer as a metabolic disease appears to be remarkably simple” [9].

When Hanahan and Weinberg introduced their six hallmarks of cancer two decades ago [10], cancers were seen as having six essential alterations in cell physiology that collectively dictated malignant growth. These were self-sufficiency in growth signals, insensitivity to growth-inhibitory signals, evasion of programmed cell death (apoptosis), limitless replicative potential, sustained angiogenesis and tissue invasion and metastasis. There was no mention of metabolism. Their second rendering of Hallmarks of Cancer [11] acknowledged that the preceding decade had introduced reprogramming of energy metabolism as an emerging hallmark. There was now a recognition that activated oncogenes such as *RAS* and *MYC* and mutant tumor suppressors like *TP53* were involved in a switch away from mitochondrial oxidative phosphorylation to cytosolic glycolysis. This rewiring of energy metabolism was first observed as a characteristic of cancer by Otto Warburg in the 1920s [12,13] and is now widely known as “the Warburg effect”. The aerobic glycolytic process produces an approximately 18-fold lower amount of ATP but permits the rechanneling of glycolytic intermediates into biosynthetic pathways that produce amino acids, nucleosides and other building blocks required for cell division. Certain cancer-associated mutations can therefore enable cancer cells to metabolize nutrients in a way that supports proliferation rather than efficient energy production [14]. The terminal metabolite in glycolysis is lactate, which is secreted by the cancer cells. Interestingly, a subpopulation of cancer cells has been described that use the lactate produced by neighboring cells as their principal energy source [15].

**Figure 1:**
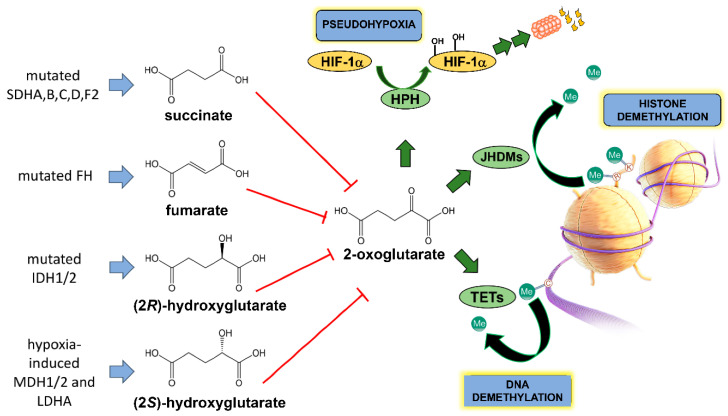
Origin of the four TCA cycle-related oncometabolites and their structural similarity to 2-oxoglutarate. SDHA/B/C/D/F2 represent succinate dehydrogenase complex subunits A–D and succinate dehydrogenase complex assembly factor 2; FH represents fumarate dehydratase; IDH1/2 represent isocitrate dehydrogenase 1 and 2; MDH1/2 represent malate dehydrogenase 1 and 2; LDHA represents lactate dehydrogenase A. HPH is HIF prolyl hydroxylase that leads to HIF-1α proteasomal degradation, JHDMs are lysine demethylases (Jumonji C domain-containing histone demethylases) and TETs are ten–eleven translocation (TET) methylcytosine dioxygenases.

The repurposing of cellular energy metabolism is by no means the only metabolic trait that cancers can express. Another metabolic idiosyncrasy that cancer cells use to their advantage is the production of so-called oncometabolites. Perusal of the literature reveals that there is no agreed concept of an oncometabolite. Many definitions are very precise and refer to the structurally similar metabolites arising from the tricarboxylic acid (TCA) cycle in cancer cells and that promote tumorigenesis and progression through similar epigenetic mechanisms. The four examples given were (2*R*)-hydroxyglutarate (D-2-hydroxyglutarate), (2*S*)-hydroxyglutarate (l-2-hydroxyglutarate), succinate and fumarate [16,17,18]. Gene mutations related to the TCA cycle enzymes fumarate hydratase (FH), succinate dehydrogenase (SDH) and isocitrate dehydrogenase (IDH) were variously discovered in cancer cells. This led to a new paradigm of oncometabolite-driven tumorigenesis, whereby mitochondrial metabolites accumulated in certain cancers and acted as oncogenic signaling molecules [19]. Some authors considered such metabolites as “bona fide oncometabolites” [19]. These oncometabolites bear a close structural similarity to another TCA cycle metabolite, 2-oxoglutarate (2-OG; 2-ketoglutarate, α-ketoglutarate) and inhibit the pleiotropic actions of 2-OG on gene regulation (Figure 1), which is an obligate cofactor in HIF prolyl hydroxylase (HPH), histone demethylation by JHDM enzymes (lysine demethylases (Jumonji C domain-containing histone demethylases)) and in demethylation of 5-methylcytosine by TET (ten–eleven translocation) dioxygenases. The oncometabolites shown in Figure 1 prevent demethylation of both histones and DNA and therefore alter the epigenetic landscape promoting tumorigenesis [17,19,20]. Other authors advanced a more generalized characterization, that of significant alterations in cellular metabolism arising from cancer-associated gene mutations [21].

In this review, we will also examine the evidence for the existence of other oncometabolites that are unrelated to the processes depicted in Figure 1. Importantly, we will describe how the tools of metabolomics have been employed to search for oncometabolites arising from perturbations of cancer cell metabolism.

## 2. Metabolomics

There are several reports on the background of metabolomics [22] and its use in biomarker definition [23,24] and the discovery of elucidation of biochemical networks and mechanisms [25]. There has also been an almost 40-fold (on average) increase per annum of reports on metabolomics cited in PubMed over the past 20 years, attesting to the explosive emergence of this field. As has been pointed out, despite the early and disparate definitions of both metabolomics [26,27] and metabonomics [28], the latter term today usually refers to studies conducted using nuclear magnetic resonance spectroscopy (NMR), whereas the former term encompasses both NMR and mass spectrometry (MS), together with other technologies [22,24]. A working definition of metabolomics would be that previously reported [22,24], “metabolomics studies the low molecular weight metabolites [e.g., <1.5 kDa] found in cells and organisms, usually through the analysis of plasma/serum, urine or cell culture medium using mainly MS or NMR technologies.” The different ways in which metabolomic investigations can be conducted has been discussed in detail by us and others [23,24]. One analytical method ideal for the separation, detection and quantitation of small molecular intermediates, such as the known oncometabolites succinate, fumarate and 2-hydroxyglutarate, is gas chromatography–mass spectrometry (GC–MS). Liquid chromatography-based methodologies, such as ultraperformance chromatography coupled with time-of-flight or Orbitrap mass spectrometry, are most commonly used in metabolomic investigations. They have the important benefits of simple or no sample preparation, high throughput and generated features (mass/charge ratios (*m*/*z*)/retention times (RT)) that run into thousands per sample analyzed, especially when the instruments are operated in both the positive and negative ion electrospray modes. That notwithstanding, translating thousands of ions that can comprise multiple types of adducts and front-end fragment ions into molecular candidates based upon *m*/*z* and RT values is not always straightforward, especially in the case of isomers. GC-MS, on the other hand, despite reduced throughput and yield of features, coupled with a need to derivatize samples to render the analytes volatile, produces diagnostic mass spectra. These spectra are generated, say, 5/s by electron ionization (EI) at 70 eV, conditions that permit comparison with standardized libraries of spectra for the purpose of compound identification. The current NIST/EPA/NIH EI–MS library contains over 350,000 spectra. We adopted a GC-MS metabolomic workflow to interrogate the small intermediary metabolite component of the plasma [29,30,31,32,33], urine [30], liver [31,34] and cultured cell [35,36] metabolomes. Others have utilized gas chromatography coupled to a time-of-flight mass spectrometer (GC–TOFMS) in cancer research [37]. It is worth noting that GC–MS methodologies were employed in the earliest metabolomics investigations, which sought to characterize the chemical composition of leaf extracts from *Arabidopsis thaliana* [27,38]. Regarding LC–MS methodologies, a large number of diverse protocols has been published for both targeted [39] and untargeted [40] approaches.

## 3. TCA Cycle Oncometabolites—Role of Metabolomics

### 3.1. Fumarate

The fumarate hydratase gene (FH) was discovered to bear an N64T mutation in an individual with a Leydig cell tumor who was part of a kindred with hereditary leiomyomatosis and renal cell cancer (HLRCC). Based on the study of other tumors, the authors concluded that some Leydig cell tumors are caused by germline FH mutations [41]. A similar situation was reported for dominantly inherited uterine fibroids, skin leiomyomata and papillary renal cell cancer with various FH mutations [42]. Using pulmonary adenocarcinoma A549 cells, FH mutation was mimicked by knockdown of FH mRNA with siRNA.

Intracellular levels of fumarate, succinate, lactate and glucose were monitored using targeted metabolomics by means of NMR. These experiments doubled the intracellular fumarate concentration, with no change in succinate but dramatic elevations in both glucose and lactate, showing that knockdown of FH was sufficient to upregulate glycolysis [43]. However, in kidney cells derived from mice in which the *Fh1* gene had been disrupted, fumarate levels rose by over 100-fold compared with kidney cells from wildtype mice. In these investigations, TCA metabolites were monitored by targeted GC–MS metabolomics, both with and without addition of either [^13^C]glucose or [^13^C]glutamine [44]. Targeted GC–MS metabolomics was also employed to analyze TCA cycle metabolites in HLRCC and other tumor samples [45]. Elevated fumarate in tumors as a result of *FH* inactivation was reported to lead to stabilization of hypoxia-inducible factors (HIFs). Under conditions of normoxia, HIFs are subject to proteasomal degradation by a mechanism involving proline 4-hydroxylation of HIFs by HIF prolyl hydroxylase. Fumarate competes with the HIF prolyl hydroxylase co-substrate 2-OG causing HIF upregulation [46] (see Figure 1). Fumarate-mediated HIF upregulation coupled with adaptation to glycolysis (see above) leads to an environment permissive for tumorigenesis [43]. This state has been termed pseudohypoxia [47] as it resembles the effects of hypoxia on HIF upregulation and tumorigenesis. It is now believed to be an extensive and cooperative network involving HIFs, mitochondrial metabolism and the Warburg effect [48]. The *FH* gene has been characterized as both a “housekeeping gene par excellence” and a tumor suppressor gene [42].

**Figure 2:**
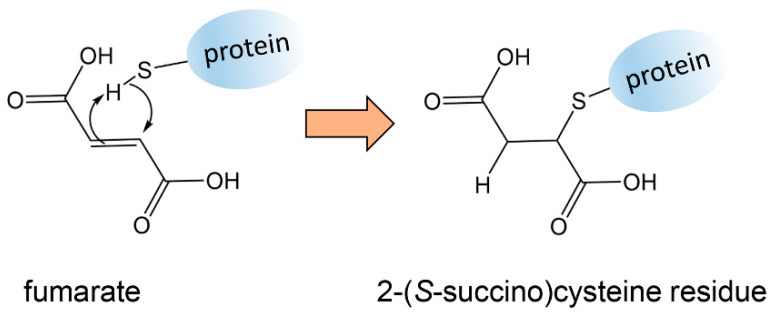
Reaction of protein thiols with elevated cellular fumarate by the Michael addition leading to succinated proteins.

Elevated cellular fumarate due to mutated *FH* has an additional metabolic consequence known as “succination”. Nucleophiles, in particular thiol groups, can add across the double bond of fumarate by the Michael addition leading to a succinate residue attached to the protein (Figure 2). Succination can have profound biological effects. For example, succination of two cysteine residues in KEAP1 results in the activation of transcription factor NRF2, which results in the transcription of genes involved in the antioxidant response [49]. It has been suggested that the cellular protective properties of NRF2 may be hijacked by cancer cells to promote cancer growth [50]. Aconitase is another TCA cycle enzyme that converts citrate stereospecifically to isocitrate. Aconitase can be succinated in FH-deficient cells through three iron/sulfur-binding cysteine residues leading to impaired aconitase activity [49]. Therefore, there are multiple metabolic effects of *FH* mutations in tumor cells, but unfortunately no untargeted metabolomics investigation appears to have been reported. The presence of succinated proteins in tumors with *FH* mutations has been detected using immunohistochemistry [51]. Other succinate-containing metabolites that were dysregulated in uterine leiomyomas with mutated *FH* were detected by LC–MS metabolomics. Specifically, N^6^-succinyladenosine and argininosuccinate were reported [52]. While it is attractive to imagine that both of these elevated metabolites were the result of the Michael addition of fumarate to the nucleophilic nitrogen atoms of adenosine and arginine, the authors advanced alternative hypotheses concerning fumarate metabolism [52].

### 3.2. Succinate

In the TCA cycle, succinate dehydrogenase (SDH) converts succinate to fumarate, which is further metabolized by FH to malate. SDH is heterotetrameric and comprises four subunits (A to D) and is the only enzyme that participates in both the TCA cycle and the respiratory electron transfer chain. The flavoprotein SDHA and iron–sulfur protein SDHB are the two catalytic subunits. SDHC and SDHD are hydrophobic membrane-anchoring subunits that are involved in binding ubiquinone in the respiratory chain. The SDHD gene (*SDHD*) was reported to bear germline mutations in hereditary paraganglioma [53]. Concurrently, *SDHC* mutations were shown to be a cause of autosomal dominant paraganglioma type 3 [54]. Subsequently, *SDHA* [55] and *SDHB* [56] mutations were said to cause pheochromocytoma and paraganglioma. Finally, *SDHAF2*, a gene that plays an essential role in the assembly of SDH and the flavination of the SDHA subunit, was found to be mutated in familial and sporadic paraganglioma and pheochromocytoma [57,58].

The SDH enzyme of the yeast *Saccharomyces cerevisiae* is structurally and functionally similar to its mammalian counterpart. The effects on the yeast metabolome of tumorigenic mutations in both human *SDHC* (*SDH3*) and *SDHD* (*SDH4*) modelled into the *S. cerevisiae* genome were explored using ^1^H NMR-based metabolomics, referred to by the authors as “metabolic footprinting” [59]. Regarding *SDHC* mutations, Arg47Lys, Arg47Cys, Arg47Glu and *SDHC* knockout (KO) had variable metabolic effects, with relatively small changes in amino acid uptake by cells relative to the wildtype (WT) but greater changes in the release of non-amino acid metabolites by mutated yeast cells into the medium relative to the WT. For example, in *SDHC*-mutated yeast, the release of succinate was ~200% of the WT, of fumarate ~50% of the WT. For *SDHD*-mutated yeast cells (Asp88Glu, Asp88Asn, Asp88Lys) and *SDHD* KO cells, the succinate and fumarate changes were less dramatic, but isobutyrate secretion was less pronounced for the KO than the WT [59]. Unfortunately, ^1^H NMR suffers from decreased sensitivity and resolution compared to mass spectrometric methodologies and it was difficult to make either a quantitative or statistical evaluation of the published data.

### 3.3. (2R)-Hydroxyglutarate

The sequencing of 13,023 genes in human breast and colorectal cancers uncovered a number of genes that were mutated at a significant frequency and that were mostly not known to be genetically altered in tumors [60]. Subsequently, this analysis was extended to the most common and lethal type of brain tumor, glioblastoma multiforme (GMB), with the sequencing of 20,661 genes. The isocitrate dehydrogenase 1 gene *IDH1* was reported to be mutated in a large number of young patients and in most patients with secondary GMB [61]. In the discovery screen, *IDH1* was mutated in five of 22 GBM tumors. Interestingly, all five had the same heterozygous point mutation leading to R132H. This R132 residue was known to bind isocitrate in the enzyme’s active site. The further functional significance of mutated *IDH1* was not known at that time. However, an untargeted metabolomic study using LC–MS was conducted that compared glioblastoma cells that were either WT for IDH1 or with the R132H, R132C, R132L or R132S mutations. Metabolomic analysis showed that *IDH1*-mutated cells had acquired a novel metabolic ability—the synthesis of (2*R*)-hydroxyglutarate (D-2-hydroxyglutarate; 2R-HG) by NADPH-dependent reduction of 2-OG [62]. Surprisingly, 2R-HG accumulated in mutant cells to a concentration of 5–35 mM. This change in metabolic activity of the R132H enzyme from oxidative decarboxylation of isocitrate to 2-OG into dehydrogenation of 2-OG to 2R-HG was rationalized by structural analysis of the enzyme’s active site [62]. In summary, tumor DNA sequencing led to the discovery of *IDH* mutations in GMB, but it was metabolomics that led to the functional significance of these mutations.

Mutations of *IDH1* were found in 188 acute myeloid leukemia (AML) samples, specifically, R132C, R132H and R132S. No R132 mutations were detected in *IDH2* [63]. It was subsequently reported that elevated concentrations of 2R-HG in AML were associated with either *IDH1* mutations or the R140Q mutation of the mitochondrial homolog IDH2. The common feature of these mutations not shared by WT cells was the production of 2R-HG. These investigators used targeted metabolic profiling by GC–MS to establish the production of 2R-HG by human embryonic kidney cells transfected with mutant *IDH* R172K [64]. Mutation of *IDH1* or *IDH2* leading to production of 2R-HG was referred to as “neomorphic enzyme activity” by these authors [64]. LC–MS-based metabolomic profiling showed that 2R-HG levels in AML cells harboring the IDH1 R132 mutation and in serum from these patients was ~50-fold higher than in WT AML [65]. Although 2R-HG appears to accumulate in cancer cells, which helps drive their proliferation, there has been a report that background physiological levels of 2R-HG can facilitate proliferation of primary fibroblasts [66]. The question has been posed, is *IDH* a tumor suppressor or an oncogene? The congruence of mutations in both *IDH1* and *IDH2* that target arginine residues involved in the binding of isocitrate together with the retention of one WT allele (no loss of heterozygosity) strongly suggests that these are oncogenes [67]. This definition would also fit an assignment of an oncometabolite for 2R-HG [62]. Mutation of *IDH1* and *IDH2* therefore represents a loss of function with respect to isocitrate metabolism and a gain of function regarding 2-OG metabolism. Mutation is not believed to occur for *IDH3*. Both IDH2 and IDH3 are found in mitochondria where they execute the canonical reaction of isocitrate to 2-OG in the TCA cycle. IDH1, on the other hand, functions in the cytoplasm and peroxisomes.

The unifying property of the three oncometabolites, succinate, fumarate and (2*R*)-hydroxyglutarate, is their ability to inhibit a class of enzymes called 2-oxoglutarate-dependent dioxygenases that function to regulate the transcription factor HIF-1α, histone demethylases and TET 5-methylcytosine hydroxylases (Figure 1).

### 3.4. (2S)-Hydroxyglutarate

Using both GC–MS and RFIC–MS (ion chromatography–MS), a metabolomics investigation of clear cell renal cell carcinoma (ccRCC), the most common kidney cancer histologic subtype, established more than 10-fold elevation of (2*S*)-hydroxyglutarate (L-2-hydroxyglutarate; 2S-HG) in tumor tissue relative to normal renal parenchyma, 2S-HG levels were greater than those of 2R-HG. Transformed cell lines of renal origin also displayed higher 2S-HG than 2R-HG [68]. These metabolomic findings led to mechanistic investigations to uncover the source of the elevated 2S-HG. An inborn error of metabolism due to loss-of-function mutations in L-2-hydroxyglutarate dehydrogenase (L2HGDH), which converts 2S-HG to 2-OG, was known to lead to urinary excretion of 2S-HG [69]. The mRNA and protein expression of L2HGDH was investigated and found to be attenuated in ccRCC. Elevated 2S-HG was also associated with impaired formation of 5-hydroxymethylcytosine (5hmC) by TET1 and TET2 [68]. Therefore, 2S-HG has similar effects on 2-oxoglutarate-dependent dioxygenases as 2R-HG (Figure 1). While low levels of L2HGDH in ccRCC tumors could explain the accumulation of 2S-HG, the question remained regarding the metabolic origin of 2S-HG. Malate dehydrogenases 1 and 2 (MDH1/2; Figure 1) were known to convert 2-OG to 2S-HG in an “off-target” reaction [70]. However, it was not known if MDH1 and 2 produced the 2S-HG observed in ccRCC tumors. However, this group subsequently reported that 2S-HG production from 2-OG by malate dehydrogenase was an adaption to hypoxia; 2S-HG inhibited electron transport and glycolysis to offset mitochondrial reductive stress caused by hypoxia [71].

The first link between an oncogene and specific metabolite production was the demonstration that lactate dehydrogenase A (LDHA) was the target of the protooncogene *MYC*. Increased LDHA was required for growth of both human and rat transformed cellular spheroids that have a hypoxic inner environment [72]. Although the production of lactate was increased in their experiments, the authors did not investigate the role of lactate *per se* in promoting anchorage-independent growth. The increase in lactate was taken as a sign of the Warburg effect [72]. However, under conditions of hypoxia, it was reported that LDHA could reduce 2-OG to selectively produce 2S-HG [73].

In the case of 2S-HG, this oncometabolite was discovered by metabolomic analysis of tumor versus unaffected tissue. This subsequently led to a better biochemical and molecular understanding of tumorigenesis. These reports demonstrate the growing interaction between intermediary metabolism and DNA biology.

## 4. Nontraditional Oncometabolites—The Role of Metabolomics

### 4.1. Sarcosine

The point has been made that while gene and protein expression have been extensively profiled in human cancers, little is known about alterations to the metabolome that characterize cancer progression [74]. Using GC–MS and LC–MS untargeted metabolomics methodologies, 1126 metabolites were profiled in 262 clinical samples related to prostate cancer. Urinary sarcosine (*N*-methylglycine) was identified as a differential metabolite that increased during prostate cancer progression to metastasis. Although various further investigations [75,76,77] linked the presence of sarcosine to prostate cancer invasion, migration and aggressiveness, a definitive mechanism for the role of sarcosine in prostate cancer was not apparent [74]. In a subsequent study, it was reported that urinary sarcosine, after a rectal digital examination, could not distinguish prostate cancer patients from those with no evidence of malignancy. Sarcosine could not be considered as a differential metabolite for prostate cancer stage, grade or aggressiveness [78,79,80]. Others also made similar conclusions regarding serum sarcosine [81]. Urinary sarcosine also failed to distinguish between prostate cancer and benign prostate hypertrophy patients together with healthy men [82]. As for the detection of prostate cancer, it was reported that the diagnostic power of sarcosine was not better than that of prostate cancer antigen PCA3 in serum or urine [83]. In contrast to early reports, a Norwegian study of 3000 prostate cancer cases and 3000 controls found that men with a high serum sarcosine concentration were at a modestly reduced prostate cancer risk [84]. Considering the most recent evaluations of sarcosine [85,86], it is best considered as a biomarker for prostate cancer, especially when combined with other biomarkers such as prostate-specific antigen (PSA). According to these findings, sarcosine does not appear to possess the hallmarks of an oncometabolite. However, a study in which human prostatic cell lines were incubated with sarcosine demonstrated that the methyl donor *S*-adenosylmethionine (SAM) was elevated, together with increases in methylation of CpG islands only in prostatic cell lines but not cell lines of nonprostate origin [87]. This report showed for the first time that sarcosine is an epigenetic modifier of prostate cells, which may contribute to its function as an oncometabolite.

### 4.2. Glycine

Using untargeted LC–MS/MS metabolomics on the NCI-60 cancer cell lines, the consumption and release of 219 metabolites from the media were profiled. Glycine consumption and the manifestation of glycine synthesis in mitochondria correlated with cancer cell proliferation rates. The unexpected finding that rapidly proliferating cancer cells had an increased reliance on glycine metabolism was not reproduced in rapidly proliferating nontransformed cells. It was postulated that glycine may be used for de novo purine nucleotide biosynthesis or that one-carbon groups derived from glycine may be used in cellular methylation reactions [88]. Moreover, glycine is part of the serine–glycine one-carbon pathway that is involved in the synthesis of the purines and pyrimidines necessary for rapid cell proliferation. In addition, methylation of DNA and histones (Figure 1) that regulate the epigenetic landscape of the cell requires the cofactor SAM. Following methyl transfer from SAM, the serine–glycine one-carbon pathway is necessary for the resynthesis of methionine [89]. Together with glutamine, a principal fuel of cancer cells [90], glycine, serine and methionine are obligatory fuels for these pathways. Of these, only methionine is an essential amino acid and therefore to satisfy its need for this amino acid, the cancer cell must obtain it from the extracellular environment. All four amino acids are imported to the cancer cell via the upregulated transporters SLC6A14 and SLC38A5 [89].

Tumor initiation cells in non-small cell lung cancer (NSCLC) were reported by transcriptomic analysis to possess highly elevated expression of glycine decarboxylase (GLDC), which was found to induce striking changes in glycolysis and glycine/serine metabolism. This led the authors to describe *GLDC* as an oncogene. Furthermore, GLDC metabolic activity was required for its tumorigenic function [91]. LC–MS-based metabolomics was used to profile HLF and 3T3 cells overexpressing GLDC, in addition to A549 lung adenocarcinoma cells with retroviral knockdown of *GLDC*. Glycolytic intermediates glucose 1-phosphate and phosphoenolpyruvate, together with the pyrimidines thymidine, deoxyuridine, thymine, cytosine and uracil, were all upregulated in the HLF and 3T3 cells. In particular, pyruvate, thymidine and thymine were downregulated in the A549 cells with *GLDC* knockdown. Supplementation with sarcosine increased proliferation of A549 cells with *GLDC* knockdown [91].

Although glycine is not a TCA cycle intermediate, it bears some of the characteristics of an oncometabolite. Specifically, it seems to be produced enzymically in cancer cells and it appears to promote cancer cell proliferation. However, a clear relationship between these two characteristics is lacking. Future research may establish glycine as a bona fide oncometabolite.

### 4.3. Hypotaurine

Using capillary electrophoresis–mass spectrometry (CE–MS), 247 metabolites were measured, of which 16 were statistically significantly decreased and four increased in GBM relative to grossly normal surrounding brain tissue. When compared across tumor grades II to IV, hypotaurine had the strongest correlation with tumor grade. Homocysteic acid (HCA), an inhibitor of cysteine sulfinic acid (CSA) decarboxylase (EC 4.1.1.29) that produces hypotaurine from cysteinesulfinate, caused arrest of U-251 glioblastoma cells [92]. Using GC–MS profiling, CSA was reported to have a > 23-fold level in glioblastomas compared with grade 2 gliomas [93,94]. As shown in Figure 1, 2-OG is the oxygen donor for the hydroxylation of HIF-1α. It was reported that hypotaurine diminished levels of hydroxylated HIF-1α in U-251 cells by inhibition of HIF prolyl hydroxylase in a concentration-dependent manner, showing that hypoxia signaling was activated by hypotaurine. It was also reported that taurine could suppress the formation of hypotaurine. When given in their drinking water to mice bearing xenotransplanted glioblastomas, taurine produced a marked growth delay of transplanted tumors [92]. The second enzyme system that can produce hypotaurine is (2-aminoethanethiol) dioxygenase (ADO; EC 1.13.11.19), which synthesizes hypotaurine from cysteamine [95]. Using CE–MS, it was reported that hypotaurine is one of the top-ranked metabolites that could distinguish glioblastomas from low-grade gliomas. There was also a strong association between expression levels of ADO and hypotaurine concentrations in tumors [92]. A recent study was reported in which ADO was abrogated using CRISPR/Cas9-mediated gene editing. This limited the proliferation of glioblastoma cells in vitro and tumor growth in vivo in a mouse model [96]. These findings strongly support the role of hypotaurine as an oncometabolite in glioblastoma and point to potential druggable targets for this tumor.

### 4.4. Lactate

Most cancer cells augment glucose and glutamine consumption to fulfill their need for rapid proliferation. In doing so, energy metabolism is deregulated away from mitochondrial oxidation of pyruvate in favor of glycolysis, the end product of which is lactate that is actively exported from the cancer cell [97]. Until comparatively recently, lactate was considered to be mere “metabolic junk”. Recently, it has been recognized that lactate that is discarded by hypoxic cancer cells and cancer-associated fibroblasts can be utilized as a fuel by well-oxygenated cancer cells that are close to blood vessels. This metabolic symbiosis is essential for the progression of fast-growing tumors [97,98]. This participation of lactate in cancer–cancer and cancer–stroma shuttles was foreshadowed by the work from 35 years ago that first described the “lactate shuttle” in relation to exercise [99,100]. Lactate has now been characterized as a “signaling oncometabolite” [101]. Lactate is sensed by the G protein-coupled receptor Gpr132 of macrophages leading to cancer cell adhesion, migration and invasion correlating with metastasis and poor prognosis in breast cancer [102]. Competition for nutrients between cancer cells and immune cells, such as tumor-associated macrophages (TAMs), arises due to hypoxic regions within the tumor. Oncometabolites such as lactate, but also succinate and 2-HG, interact with TAMs to alter their phenotype and enhance tumor progression [103].

The question is, what role has metabolomics played in establishing lactate as an oncometabolite? In a report of 2-HG quantitation in human gliomas using high-field ^1^H-MRS, lactate was found to be elevated fivefold in the IDH1R132H and IDH2R172K mutant brain tumor tissue compared with healthy brain tissue [104]. This association with 2-HG in mutated glioma was not in itself evidence that lactate was an oncometabolite, but provided a promising sign. Lactate has been described as a cancer-associated metabolite biomarker in colorectal cancer [105] and thyroid cancer [106]. High-resolution magic-angle spinning ^1^H NMR has been employed in a metabolomics investigation of malignant thyroid tissue [107] and of prostate cancer [108], and in both cases lactate was the predominant biomarker.

In summary, several reports have established a role and a mechanism for lactate as an oncometabolite. The production of lactate by tumors was first observed by Warburg [12,13] but its characterisation as an oncometabolite has been aided by its detection in several types of cancer by metabolomics.

### 4.5. Kynurenine

Kynurenine is a metabolite of the essential amino acid tryptophan (and the least abundant amino acid in most proteins). As a consequence, most cellular tryptophan is converted to serotonin or to kynurenine, the latter long known to be the starting point of the pathway leading to de novo nicotinic acid and NAD synthesis [109]. It has been reported that the oncogenic transcription factor MYC mediates enhanced expression of the tryptophan importers SLC1A5 and SLC7A5 together with the enzyme AFMID that synthesizes kynurenine from *N*-formylkynurenine [110]. By activating the transcription factor AHR, kynurenine regulates growth promoting genes in cancer cells [111]. Interestingly, increased flux through the kynurenine pathways will lead to elevated nicotinic acid. NADH and NADPH [112] have recently been reported as being overexpressed in tumor cells with mutant p53 [113].

Kynurenine appears to be a good candidate for an oncometabolite. Moreover, the discovery of enhanced tryptophan cellular uptake and metabolism in the kynurenine pathway was established using UPLC–TQMS metabolomics [110].

### 4.6. Methylglyoxal

One of the consequences of the Warburg effect is that augmented cellular glycolytic flux can lead to increased formation of methylglyoxal (MGO), a principal precursor of advanced glycation end products (AGEs). As a result, enhanced protein glycation may contribute to a number of pathologies, including cancer [114]. MGO irreversibly modifies an estimated 1 to 5% of proteins at arginine residues, forming the MGO-derived argpyrimidine [115] and hydroimidazolone [116] AGEs. The accumulated evidence suggests that MGO-modified proteins connect diabetes with cancer [117] and are associated with several cancers [118]. By silencing the detoxication of MGO by glyoxalase 1, it was reported that MGO favored nuclear localization of the transcriptional coactivator YAP (Yes-associated protein) [119] and inactivation of the Hippo tumor suppressor pathway [120] in breast cancer cells, thereby promoting proliferation [121]. A metabolomic investigation has been reported in which HeLa cervical cancer cells were profiled by LC–MS at different phases of the cell cycle, and 921 metabolites were detected. The authors stated that MGO in particular was found to oscillate in concentration according to the phase of the cell cycle, with higher concentrations in G_1_ than in SG_2_M [122]. It had earlier been reported that MGO activated the checkpoint kinases Chk1 and Chk2 in an AGE-dependent manner in human embryonic kidney HEK 293 cells [123].

The potential role of MGO as an oncometabolite appears to involve the formation of AGEs and deserves further investigation.

### 4.7. Miscellaneous

One example of a putative oncometabolite based upon a single study is (2*R*,3*S*)-dihydroxybutanoic acid (2,3-DHBA). A GC–MS-based metabolomics investigation into AML patients with and without *IDH1* and *IDH2* mutations was reported. As expected, 2-HG was highly statistically significantly elevated in plasma of patients with IDH1R132 and IDH2R140 neomorphic enzymes. Unexpectedly, another plasma metabolite, 2,3-DHBA, was similarly elevated and highly correlated with plasma 2-HG levels. When ROC analysis was conducted, 2,3-DHBA was a superior biomarker (80% specificity; 87.3% sensitivity) for IDH1/2 mutation in AML compared to the oncometabolite 2-HG (80% specificity; 63.8% sensitivity) [33].

**Figure 3:**
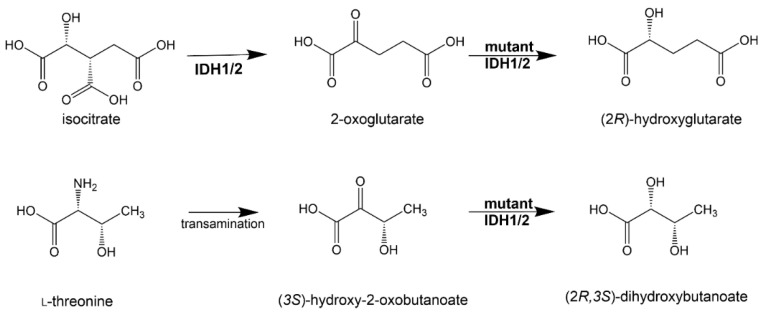
Structural relationship between the putative oncometabolite (2*R*,3*S*)-dihydroxybutanoate, oncometabolite (2*R*)-hydroxyglutarate, isocitrate and 2-oxoglutarate.

It has been proposed that 2,3-DHBA is synthesized by mutant IDH1 and IDH2 similarly to the synthesis of 2R-HG from 2-OG. Moreover, we have also suggested that the precursor of 2,3-DHBA in this reaction is (3*S*)-hydroxy-2-oxobutanoate formed by the transamination of the amino acid L-threonine (Figure 3) [33]. The properties of 2,3-DHBA with respect to cancer cell proliferation and its epigenetic regulation are yet to be established.

A few reports have nominated oncometabolites that are supported by little evidence. In discussing the role of a diet, gut microbiota and metabolism on the incidence of colorectal carcinoma, digestion of dietary red meat and the metabolism of primary bile acids by the gut microbiota were said to produce hydrogen sulfide (H2S) and secondary bile acids, such as deoxycholic acid (DCA). H2S and DCA were characterized as oncometabolites in colorectal carcinoma [124]. Published metabolomic data were reviewed for investigations into thyroid cancer, whereby cancerous cells had been compared to their “counterpart noncancerous cells”. Metabolites that differed between these two sets of samples were characterized by these authors as “oncometabolites”, suggesting that metabolites found more commonly in cancer cells are oncometabolites [106], an interpretation with which we would disagree. The authors relied on the report of David Wishart et al. [105] who, in addition to the oncometabolites discussed above, listed asparagine (leukemia), choline (prostate, brain, breast cancer) and polyamines (most cancers). The principal criterion for inclusion in their list was “endogenous metabolites that either initiate or sustain tumor growth and metastasis”.

Table 1 lists the known and putative oncometabolites whose characteristics have been defined by metabolomics.

## 5. Rewiring Cancer Metabolism as a Therapeutic Strategy

Ninety years ago, Warburg found that cancer relies on unusual metabolic pathways to fuel its rapid growth [12,13]. This concept was adopted by Agios Pharmaceuticals when they began to research *IDH* mutations in glioma and AML [128] and then reported that cancer-associated *IDH1* mutations produced 2R-HG, which they identified as an oncometabolite [62]. Agios developed a first-in-class drug AG-221 (enasidenib) that specifically targeted AML harboring oncogenic *IDH2* mutations [129]. Enasidenib was granted regulatory approval by the FDA on 1 August 2017 for the treatment of adult patients with relapsed or refractory acute myeloid leukemia with an isocitrate dehydrogenase-2 (IDH2) mutation as detected by an FDA-approved test [130]. The efficacy of the drug is based upon inhibition of the gain of function associated with production of 2R-HG by cancer cells. Decreasing levels of the oncometabolite attenuated the epigenetic dysregulation and removed the block on cellular differentiation [129]. While a typical cancer cell is replete with mutations, no other gain-of-function mutations that lead to oncometabolites have so far been described [130].

In the case of succinate and fumarate, there have been drugs developed in relation to SDH and FH. However, a metabolomic screen of hPheo1 pheochromocytoma cells and SDHB knockdown hPheo1 cells together with cancer tissues with and without SDHx mutations revealed that the polyamines spermidine and spermine were significantly elevated in relation to mutated succinate dehydrogenase. Accordingly, polyamine pathway inhibitors inhibited the growth of hPheo1 cells in vitro as well as mouse xenografts [131]. These findings open up a new therapeutic avenue for pheochromocytomas bearing SDHx mutations. However, they are not based upon rewiring of an oncometabolite pathway.

## 6. Conclusions

Mutations in oncogenes and tumor suppressor genes were long believed to initiate the principal pathways to tumorigenesis. Nevertheless, a supplementary pathway exists that involves the metabolic reprogramming of cancer cells. Mutations that lead to inactivated enzymes involved in intermediary metabolism can cause cellular accumulation of small molecules that trigger or amplify oncogenic pathways. Such small molecules are now known as oncometabolites. The link between cancer mutations and the cancer cell metabolic phenotype has been studied extensively using metabolomics. Mutations in fumarate hydratase, succinate dehydrogenase and isocitrate dehydrogenase together with hypoxia-driven promiscuous substrate usage by both lactate dehydrogenase A and malate dehydrogenase 2 lead to the formation of oncometabolites. As a result, the modified metabolic phenotypes can drive cancer cell proliferation and progression. Metabolomic investigation of cancer metabolic phenotypes has led to the understanding that cancer cell accumulation of the oncometabolites fumarate, succinate, (2*R*)-hydroxyglutarate or (2*S*)-hydroxyglutarate propels tumorigenesis. The affected pathways largely involve TCA cycle metabolites. There is a second group of metabolites involved in amino acid metabolism, which can best be described as putative oncometabolites. These include sarcosine, glycine, hypotaurine and (2*R*,3*S*)-dihydroxybutanoate. Further research is required to establish these as oncometabolites. Metabolomic analysis has been central to the discovery and definition of both the established and putative oncometabolites. Metabolic rewiring of cancer cells permits them to escape from housekeeping metabolic duties and switch to the synthesis of metabolic building blocks required for proliferation. There is only one known example of a gain-of-function mutation unique to cancer tissues and this has impeded the development of drugs that can oppose the effects of oncometabolites without affecting healthy cells. It is nevertheless clear that metabolomics will continue to play a significant role in the study of cancer.

## Figures and Tables

**Table 1:** Known and putative oncometabolites defined by metabolomics.

Oncometabolite	Tumors	Metabolomic Contribution	Strength of Evidence	Reference
Fumarate	HLRCC	Linked *FH* mutation to TCA and glycolytic metabolites	+++	[43,44,45]
Succinate	Hereditary paraganglioma Pheochromocytoma	Linked *SDH* mutations to succinate and other metabolites	+++	[59]
(2*R*)-Hydroxyglutarate	Glioblastoma multiforme Acute myeloid leukemia	Linked *IDH1* and *IDH2* mutations to 2R-HG	+++	[33,62,64,65]
(2*S*)-Hydroxyglutarate	Clear cell RCC	Linked L2HGDH activity in tumors to 2S-HG	+++	[68]
Lactate	Several cancers	Signaling molecule contributing to proliferation, migration, invasion, angiogenesis, immune system escape and resistance to therapy	+++	[97,101,125,126]
Kynurenine	Colon cancer	Activation of transcription factor AHR that regulates growth-promoting genes	+	[110,111]
Methylglyoxal	Breast and colorectal cancer	AKT activation through PI3K/mTORC2 and Hsp27 regulation	+	[118,127]
Sarcosine	Prostate cancer	Variable reports of linkage of sarcosine to prostate cancer; no clear mechanism; potential biomarker	±	[74,75,76,77,78,79,80,81,82,83,84,85,86]
Glycine	NCI-60 cell lines	Linked glycine metabolism to rapidly proliferating cancer cells; postulated mechanism involving glycine decarboxylase (GLDC); cells overexpressing GLDC → ↑glycolytic intermediates and ↑pyrimidines	+	[88,91]
Hypotaurine	Glioblastoma multiforme	Established role for hypotaurine in glioblastoma multiforme; correlative and mechanistic data point to hypotaurine as an oncometabolite	++	[92]
(2*R*,3*S*)-Dihydroxybutanoate	Acute myeloid leukemia	Found in plasmas of mutant IDH1/2, 2,3-DHBA is greater than WT IDH1/2 and strongly correlated with 2R-HG; 2,3-DHBA is a better biomarker for mutated IDH than classical oncometabolite 2R-HG	±	[33]

+++, ++, and + indicate strength of evidence that is high, intermediate or low, respectively. ± indicates strength of evidence that is currently equivocal.
